# CaMKK2 Expression Correlates with High-Risk CLL Biology, and Pharmacologic Inhibition Is Associated with Reduced Leukemic Cell Survival and Nurse-like Cell Support In Vitro

**DOI:** 10.3390/cells15141294

**Published:** 2026-07-20

**Authors:** Shekeab Jauhari, Alicia D. Cooper-Volkheimer, Vini Verma, Dilber Gökçe Kaplan, Fahmin Basher, J. Brice Weinberg, Nelson J. Chao, Luigi Racioppi

**Affiliations:** 1 Division of Hematological Malignancies and Cellular Therapy, Department of Medicine, Duke University, Durham, NC 27707, USA; shekeab.jauharimd@gmail.com (S.J.); alicia.volkheimer@duke.edu (A.D.C.-V.); dilbergokcekaplan@gmail.com (D.G.K.); fahmin.basher@duke.edu (F.B.); nelson.chao@duke.edu (N.J.C.); 2 Division of Hematology, Department of Medicine, Duke University, Durham, NC 27707, USA; 3 Department of Medicine, Durham VA Medical Center, Durham, NC 27707, USA; 4 Department of Molecular Medicine and Medical Biotechnology, University of Naples Federico II, 80131 Naples, Italy

**Keywords:** chronic lymphocytic leukemia, CaMKK2, calcium signaling, IGHV-unmutated CLL, nurse-like cells, tumor-associated macrophage, tumor microenvironment

## Abstract

**Highlights:**

**What are the main findings?**

- High CaMKK2 expression in CLL cells is associated with adverse clinical and molecular features and earlier need for treatment.
- Pharmacologic inhibition of CaMKK2 reduces CLL cell viability and alters nurse-like cell/macrophage support in vitro.

**What are the implications of the main findings?**

- CaMKK2 may contribute to biological heterogeneity across CLL subsets.
- Targeting CaMKK2 may represent a strategy to modulate both leukemia cells and the supportive tumor microenvironment, warranting further investigation.

**Abstract:**

Background/Objectives: Chronic lymphocytic leukemia (CLL) is characterized by clinical and biological heterogeneity shaped by intrinsic signaling programs and microenvironmental interactions. Established biomarkers, including IGHV mutation status and TP53 alterations, provide important clinical and molecular information, but do not fully capture the diversity of pathways that sustain leukemic cell fitness. Aberrant calcium signaling contributes to leukemic survival; however, the clinical relevance of Ca^2+^/calmodulin-dependent protein kinase kinase 2 (CaMKK2), a calcium-responsive kinase, has not been defined. This study evaluated CaMKK2 as a candidate marker associated with high-risk disease biology and a pathway of interest for further study. Methods: CaMKK2 expression was quantified in purified CD19^+^ CLL cells from a clinically annotated cohort balanced by immunoglobulin heavy chain variable region (IGHV) mutation status. Associations with time to treatment and overall survival were analyzed. Functional relevance was assessed by pharmacologic inhibition of CaMKK2 in primary CLL cells using metabolic (MTS) and apoptosis (Annexin V/PI) assays. Correlations between CaMKK2 expression and inhibitor sensitivity were determined. The impact of CaMKK2 inhibition on nurse-like cell (NLC) differentiation and macrophage-mediated leukemic support was evaluated in ex vivo culture systems. Results: Elevated CaMKK2 expression was enriched in IGHV-unmutated CLL and associated with shorter time to treatment and inferior overall survival. Pharmacological inhibition of CaMKK2 was associated with reduced primary CLL viability in a dose-dependent manner and increased Annexin V/PI-defined total cell death with sensitivity correlating with CaMKK2 expression levels. Inhibition also attenuated CD163^+^ macrophage polarization and impaired NLC-mediated support of leukemic cells. Conclusions: CaMKK2 expression is associated with IGHV-unmutated, high-risk CLL biology. Pharmacologic inhibition of CaMKK2 was associated with reduced leukemic cell viability and altered macrophage phenotypes in ex vivo systems. These findings are exploratory, derived from a limited cohort, and support further investigation into the role of CaMKK2 in CLL biology, but do not establish independent prognostic value or direct on-target causality.

## 1. Introduction

Chronic lymphocytic leukemia (CLL) is a biologically heterogeneous B-cell malignancy characterized by progressive accumulation of CD5^+^CD19^+^ B cells in blood and lymphoid tissues. Clinical behavior ranges from indolent disease to rapidly progressive forms requiring early treatment [1,2]. Among established prognostic features, immunoglobulin heavy chain variable region (IGHV) mutation status remains a central determinant of outcome, with IGHV-unmutated CLL associated with enhanced B-cell receptor (BCR) signaling and inferior clinical prognosis [3,4].

CLL progression reflects both intrinsic leukemic cell programs and microenvironmental support. Within lymph node and marrow niches, stromal elements, T cells, and tumor-associated macrophages, including nurse-like cells (NLCs) [5,6], deliver cytokines, chemokines, and direct contact signals that reinforce BCR-associated pathways, sustain metabolic fitness, and limit apoptosis [7]. These niche-derived signals can attenuate responses to targeted therapies and contribute to minimal residual disease [8,9,10]. Thus, effective therapeutic strategies may require disruption of both leukemic survival pathways and macrophage-mediated niche protection.

Aberrant calcium signaling is a defining feature of CLL biology [11]. BCR activation promotes sustained intracellular Ca^2+^ flux through store-operated calcium entry (SOCE), mediated by ORAI1 and STIM channels [12]. Calcium-dependent signaling supports NFAT activation, metabolic adaptation, and apoptosis resistance [13]. While proximal BCR signaling has been successfully targeted with BTK inhibitors, emerging evidence suggests that downstream calcium-dependent programs may persist despite inhibition of upstream nodes [14]. The molecular effectors that couple SOCE to metabolic resilience in CLL remain incompletely defined.

Ca^2+^/calmodulin-dependent protein kinase kinase 2 (CaMKK2) is a serine/threonine kinase activated by Ca^2+^/calmodulin that regulates AMPK and related metabolic stress-response pathways [15]. CaMKK2 has been implicated in multiple malignancies, where it promotes tumor cell survival, macrophage polarization, and tumor progression [16,17,18,19,20,21,22]. In myeloid cells, CaMKK2 contributes to tumor-associated macrophage differentiation and immunosuppressive programming [23,24]. Recent work further links CaMKK2 to matrix-mediated mechanosensory signaling and AKT activation, positioning it at the convergence of calcium signaling and biomechanical adaptation [25]. However, the role of CaMKK2 in CLL, particularly in coordinating leukemic survival with macrophage-mediated niche support, has not been defined.

We hypothesized that CaMKK2 may function as a calcium-responsive signaling node linking leukemic cell survival with macrophage-mediated microenvironmental interactions. To explore this, we examined CaMKK2 expression in clinically annotated CLL cohorts and evaluated associations with disease features. We also assessed the effects of pharmacologic inhibition on primary CLL cells and macrophage-like systems. Our aim was to define whether CaMKK2 expression is associated with high-risk disease biology and whether modulation of this pathway impacts leukemic and microenvironmental phenotypes in vitro.

## 2. Materials and Methods

CLL patient blood samples. Peripheral blood samples were obtained from CLL patients under Institutional Review Board-approved protocols Pro00011267, approved on 29 August 2008, and continuing review, approved on 8 December 2024, at Duke University and the Durham VA Medical Center.

Patients were either treatment-naïve or had not received therapy within two years prior to peripheral blood collection. Available clinical and laboratory variables included age, sex, date of diagnosis, Rai stage, treatment history, cytogenetic abnormalities assessed by fluorescence in situ hybridization (FISH), and molecular prognostic markers, including IGHV mutation status, ZAP-70 expression, and CD38 expression. While the cohort was intentionally balanced by IGHV mutation status to enable biologically relevant comparisons, other clinical variables, including Rai stage and treatment history, were not uniformly matched across groups and may influence outcome analyses. Clinical and laboratory characteristics of the patient cohorts are summarized in Supplementary Tables S1 and S2, and in the corresponding figure legends.

For analyses of CaMKK2 expression and clinical outcomes, a cohort of 40 patients was assembled and intentionally balanced by IGHV mutation status (20 IGHV-mutated and 20 IGHV-unmutated cases). IGHV status represents a major biological and prognostic determinant in CLL, reflecting differences in B-cell receptor signaling strength and disease aggressiveness [26]. This balanced design minimized subtype-driven bias, enabled direct comparison between risk groups, and permitted evaluation of CaMKK2 associations independent of cohort imbalance.

B lymphocytes were isolated from whole blood by negative selection using the RosetteSep™ Human B Cell Enrichment Cocktail (STEMCELL Technologies, Vancouver, BC, Canada) and cryopreserved at −80 °C for RNA isolation and quantitative RT-PCR (qRT-PCR) analysis, as described in the corresponding section. Of the 40 cases initially selected, 33 were evaluable for *CaMKK2* expression based on predefined quality control criteria for RNA integrity and qRT-PCR performance. Samples failing to meet these criteria were excluded from analysis (Supplementary Tables S1).

Generation of nurse-like cells (NLCs). NLCs were generated from CLL Peripheral blood mononuclear cells (PBMCs) as previously described [6]. Briefly, PBMCs were isolated from DMSO-cryopreserved CLL PBMCs by density-gradient centrifugation using Ficoll-Paque (Sigma-Aldrich, St. Louis, MO, USA). PBMCs were resuspended in RPMI 1640 supplemented with 15% fetal calf serum and 1% penicillin–streptomycin and glutamine. PBMCs were then plated at high density (2 × 10^7^ cells/mL) in tissue culture-treated plates (Corning, Corning, NY, USA) and maintained at 37 °C in a humidified 5% CO_2_ atmosphere to allow differentiation of adherent NLCs. Where indicated, cultures were treated at the time of plating (day 1) with the CaMKK2 inhibitor STO-609 (5 µM; Tocris Bioscience, Bristol, UK) or an equal volume of vehicle (Veh; DMSO). Supplemental doses of STO-609 (2.5 µM) or vehicle were added on days 5 and 10. To minimize drug accumulation while preserving early adherent precursors, partial medium changes were performed during treatment. After 14 days, non-adherent CLL-enriched cells were collected for analysis, and adherent cells were extensively washed with PBS to remove residual non-adherent cells. Adherent NLCs were then imaged directly by optical microscopy and subsequently detached using Macrophage Detachment Solution (PromoCell GmbH, Heidelberg, Germany), according to the manufacturer’s instructions, and used for flow-cytometric phenotyping, or processed for RNA isolation and qRT-PCR, as indicated. These culture conditions are designed to model macrophage–CLL interactions; however, prolonged ex vivo culture may influence macrophage differentiation states and associated signaling pathways.

Cell lines and preparation of tumor-conditioned medium (TCM). To model B-cell malignancy-derived factors within the tumor microenvironment, we used tumor-conditioned medium from human B cell tumor cell lines including OPM2, a multiple myeloma cell line derived from a patient with plasma cell leukemia and commonly used as a model of aggressive plasma cell malignancy [27]; BJAB from Burkitt lymphoma [28]; and SU-DHL-4, a diffuse large B-cell lymphoma originally derived from the peritoneal effusion of a 38-year-old male with non-Hodgkin lymphoma [29]. Briefly, tumor cells growing in logarithmic growth phase with viability > 95% were harvested, washed twice with PBS, and resuspended in fresh complete medium at a density of 1–2 × 10^6^ cells/mL. Cultures were incubated at 37 °C in a humidified 5% CO_2_ atmosphere for an additional 24–48 h. Cell supernatants were then collected, centrifuged to remove cells, filtered through a 0.22 µm membrane, aliquoted, and stored at −80 °C until use.

Generation of monocyte-derived macrophages (MDMs) from healthy donor leukocytes. Leukocytes from healthy donors were obtained from the Gulf Coast Regional Blood Center as de-identified commercial human blood samples. Donor leukocytes were derived from single units of whole blood by density-gradient centrifugation. Donors were ≥16 years of age and met standard eligibility criteria for blood donation. All identifying information was removed prior to sample transfer, and samples were approved for in vitro research use; therefore, this protocol was determined to be IRB-exempt. Monocyte-derived macrophages were generated using a modified version of a previously described protocol [30]. Briefly, PBMC were resuspended in Monocyte Attachment Medium (PromoCell) and seeded into 12-well tissue culture plates at a density of 2–4 × 10^6^ cells per well. Cells were incubated at 37 °C in a humidified 5% CO_2_ atmosphere for 1–2 h to allow monocyte adherence. Non-adherent cells were removed by extensive washing and adherent monocyte-enriched cells were cultured in macrophage differentiation medium (RPMI 1640 supplemented with recombinant human M-CSF 20 ng/mL; PeproTech, Cranbury, NJ, USA), in the absence or presence of tumor-conditioned media (TCMs; 50% *v*/*v*). STO-609 (5 µM) or an equal volume of vehicle (DMSO) was added at the time of plating (day 1). On day 3, culture media were partially replaced with fresh macrophage differentiation medium containing TCM or regular medium, with STO-609 or vehicle, corresponding to initial treatment conditions. After 6 days of differentiation, cultures were extensively washed with PBS, and adherent MDM were detached as described for NLC preparation. MDM immunophenotype was assessed by flow cytometry, and aliquots of cells were processed for quantitative RT-PCR analysis.

MTS and Annexin V/PI assays. Primary CD19^+^ CLL cells (0.25 × 10^6^/well) were cultured in a 96-well plate in 0.1 mL of Hybridoma SFM (Gibco, Grand Island, NY, USA), as described [31,32,33]. Cells were treated with increasing concentrations of the CaMKK2 inhibitors STO-609 [34] (Tocris, Bioscience, Bristol, UK), CC-8977 [35] (Small Molecule Synthesis Facility Duke University), SGC-CaMKK2-1 (Sigma) [36], or an equal volume of vehicle (DMSO). Cells were then incubated at 37 °C in a humidified 5% CO_2_ atmosphere, and CLL viability was assessed at 72 h using two orthogonal approaches. Cytotoxicity assays were performed using the MTS [3-(4, 5-dimethylthiazol-2-yl)-5-(3-carboxymethoxyphenyl)-2-(4-sulfophenyl)-2H-tetrazolium] assay (CellTiter 96 Aqueous One Solution Cell Proliferation Assay; Promega Corporation, Madison, WI, USA), as described [37]. Briefly, MTS reagent was added to cell culture media and incubated for four hours before cell lysis by 1% SDS. Absorbance at 490 nm was measured using a microplate reader SPECTROstar Nano microplate reader, BMG LABTECH, Ortenberg, Germany). The percentage of viable cells was determined by comparing the absorbance in drug-treated cells to the absorbance in vehicle-treated cells and to calculate EC50 values. In parallel, apoptosis/cell death was quantified by flow cytometry using Annexin V-FITC (BD Biosciences, San Jose, CA, USA) and propidium iodide (PI; Sigma, St. Louis, MO, USA) staining; total Annexin V^+^ events (PI^−^ and PI^+^ populations) were quantified across the same concentration range.

Bright-field microscopy and ImageJ particle analysis. After 14 days of CLL-PBMC culture, non-adherent cells were removed, and wells were extensively washed with PBS to eliminate residual suspension cells. Adherent cells were imaged by bright-field optical microscopy using an Axiovert 200 microscope (Carl Zeiss Microscopy, Thornwood, NY, USA) and identical acquisition settings across experimental conditions. For quantitative analysis, images were imported into ImageJ (NIH), and adherent structures were segmented using a consistent preprocessing and thresholding workflow applied uniformly across all images within an experiment. Particle abundance was quantified using the ImageJ (version 1.54x) Analyze Particles function and reported as particle number per unit area (particles/mm^2^).

NLC–CLL co-culture assay. NLC from CLL PBMC were generated in the presence of vehicle or STO-609 (NLC-Veh and NLC-STO, respectively) as described above. After 14 days, CD19^+^ CLL were purified from the vehicle-treated groups. NLC-adherent cells from Vehicle and STO-609 groups were extensively washed to avoid drug carryover, detached, and dispensed in 48-well plates at 1 × 10^5^ cells/well. CD19^+^ CLL cells (0.25 × 10^5^/well) were then cultured either alone (“None”) or co-cultured with autologous NLC-Veh or NLC-STO. At the indicated time points, non-adherent cells were collected, stained with CD19, and cell viability was quantified by flow cytometry as live CD19^+^ events.

Flow cytometry. Cells were harvested at the indicated time points, washed in PBS containing 2% FCS, and stained with fluorochrome-conjugated antibodies for surface markers, with inclusion of a fixable viability dye to exclude dead cells. All antibodies were used following manufacturer’s instructions. To assess CLL yield and viability, non-adherent cells were collected and stained with PE anti-human CD19 (clone HIB19, BioLegend, San Diego, CA, USA) and live fixable dye (Zombie Near-IR; BioLegend). Adherent cells (NLC or MDM) were gently detached from wells using Monocyte Detachment Media (PromoCell), stained with Ig blocker, followed by saturating amounts of the following antibodies for 30 min at 4 °C: AF488 anti-human HLA-DR (clone L234, BioLegend), PerCP/Cy5.5 anti-human/mouse CD11b (clone M1/70; BioLegend) or PE anti-human CD14 (clone HCD14, BioLegend) and live fixable dye (Zombie Near-IR; or Zombie yellow, BioLegend). Cells were fixed in 4% paraformaldehyde (PFA, Sigma), permeabilized with Tween-20, re-stained with PE/Cy7 anti-human CD68 (clone eBioY1/82A, eBioScience, San Diego, CA, USA) and APC anti-human CD163 (clone GHI/61 eBioScience). All antibodies and staining procedures were performed according to manufacturer’s instructions. Unstained and single-stained controls were used for instrument setup and compensation, and fluorescence-minus-one (FMO) controls (with isotype controls as needed) were used to define gating thresholds. Cells were acquired with a BD FACSCanto cytometer (San Jose, CA, USA) and analyzed using FloJo 10.10.0 software.

Gene expression analysis. Following PBMC culture, wells were washed and adherent cells were imaged as described above. Adherent cells were lysed directly in Buffer RLT (Qiagen, GmbH, Hilden, Germany) and collected by gentle scraping. Lysates were homogenized using QIAshredder columns (Qiagen), and total RNA was purified using the RNeasy Mini Kit (Qiagen) according to the manufacturer’s instructions. cDNA was synthesized from purified RNA using the SensiFAST cDNA Synthesis Kit (Bioline, London, UK). Quantitative RT-PCR was performed using SYBR Green master mix, gene-specific forward and reverse primers (Integrated DNA Technologies, Coralville, IA, USA), and cDNA template on an Applied Biosystems™ QuantStudio™ 6 Flex Real-Time PCR System. Reactions were performed in technical duplicates or triplicates, as permitted by available sample material. Amplification specificity was assessed by melt-curve analysis, and reactions with non-specific amplification or failed amplification were excluded. Relative expression was calculated using the 2^−ΔCt^ method after normalization to ACTB. Primer sequences are listed in Supplementary Table S3. We note that ACTB was used as the sole reference gene, which represents a limitation of the qRT-PCR analysis.

Statistical analysis. Statistical analyses were performed using GraphPad Prism version 11.0.2 (GraphPad Software, Boston, MA, USA). Normality was assessed using Shapiro–Wilk testing and data distribution was not assumed to be normal. Overall survival and time to treatment were analyzed by Kaplan–Meier methods and compared using the log-rank (Mantel–Cox) test. For the independent validation cohort, survival analysis was performed using the SurvExpress platform [38]. Continuous Cox proportional hazards modeling was explored but not reported because the limited number of outcome events resulted in unstable model estimates and non-estimable hazard ratios. For primary CLL drug-sensitivity assays, MTS dose–response curves were fit by nonlinear regression to derive EC50 values. Associations between CaMKK2 expression and STO-609 potency, and between metabolic inhibition and Annexin V-defined cell death, were assessed using Spearman rank correlation. For paired analyses of matched patient samples, statistical significance was assessed using two-tailed paired tests. For NLC co-culture experiments, technical replicates were averaged within each patient, and paired comparisons were performed across independent biological replicates using two-tailed paired tests. For tumor-conditioned media experiments, differences across conditions were evaluated by ANOVA with multiple-comparisons testing (GraphPad Prism version 11.0.2), including planned comparisons of vehicle versus STO-609 within each conditioned-media condition. Unless otherwise stated, all tests were two-sided.

## 3. Results

**Figure 1:**
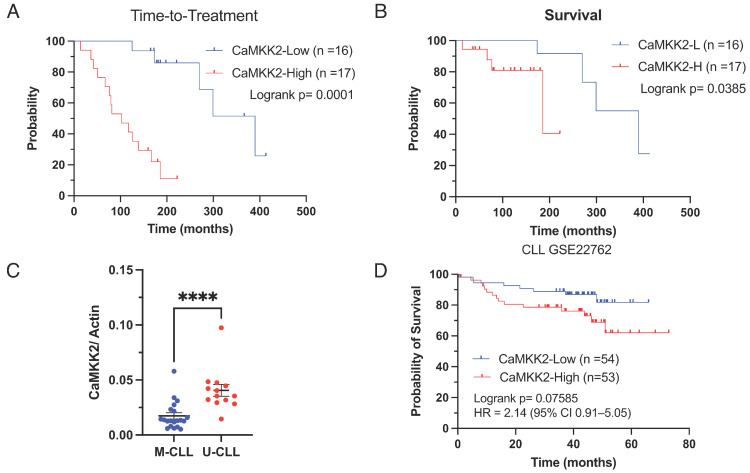
CaMKK2 expression is associated with high-risk biology and exploratory survival differences in CLL. (**A**,**B**) Kaplan–Meier analyses of time to treatment (**A**) and overall survival (**B**) for CLL patients stratified by *CaMKK2* expression using a median split (CaMKK2-Low, *n* = 16; CaMKK2-High, *n* = 17). Tick marks indicate censored observations. *p* values were calculated using the log-rank (Mantel–Cox) test. (**C**) CaMKK2 mRNA expression in cryopreserved CD19^+^ CLL cells obtained from the Duke CLL biorepository, quantified by qRT-PCR and normalized to ACTB. Samples are grouped by IGHV mutation status (IGHV-mutated [M-CLL] vs. IGHV-unmutated [U-CLL]). Each symbol represents one patient; horizontal bars indicate mean ± SEM. Statistical significance was assessed by two-tailed Mann–Whitney test (**** *p* < 0.0001). (**D**) Independent cohort validation using the publicly available CLL dataset reported by Herold et al. (GSE22762). Overall survival was analyzed using the SurvExpress platform, stratifying cases into CaMKK2-high versus CaMKK2-low groups. Log-rank *p* value and hazard ratio (HR) with 95% confidence interval (CI) are indicated. Cohort size and imbalance in clinical covariates, including Rai stage, should be considered when interpreting survival differences.

Elevated CaMKK2 expression is associated with IGHV-unmutated, high-risk CLL biology in an exploratory cohort. To evaluate whether CaMKK2 expression is associated with high-risk CLL biology in the context of established prognostic factors, we analyzed clinically annotated CLL biorepository specimens available at the Division of Hematological Malignancies and Cellular Therapy (Duke University). We selected 40 cryopreserved peripheral blood CLL samples, balanced a priori by IGHV mutation status (20 IGHV-mutated; 20 IGHV-unmutated), and quantified *CaMKK2* mRNA in purified CD19^+^ CLL cells by qRT-PCR. Cohort characteristics are summarized in Supplementary Table S1. Because CaMKK2 quantification required adequate RNA and complete clinical linkage, seven samples were excluded due to insufficient RNA quality or qRT-PCR performance, yielding 33 evaluable cases. Patients were stratified using the cohort median of *CaMKK2* expression (high versus low; group sizes shown in the plots), a pragmatic and unbiased threshold that enables balanced comparison without data-driven optimization. Stratification by median expression was used as an exploratory approach to assess associations with clinical outcome. Using this cutoff, CaMKK2-high status was associated with significantly shorter time to treatment (log-rank *p* = 0.0001; Figure 1A) and inferior overall survival (log-rank *p* = 0.0385; Figure 1B), supporting an association with adverse clinical features in this cohort; however, these findings should be interpreted as exploratory given the limited sample size and potential imbalance in clinical covariates. *CaMKK2* expression, normalized by *ACTB*, was significantly higher in IGHV-unmutated compared with IGHV-mutated CLL (Figure 1C). Furthermore, the Duke cohort recapitulated canonical IGHV-linked biology, with higher CD38 and ZAP-70 in IGHV-unmutated cases (Supplementary Figure S1). An attempted continuous Cox model was unstable and non-estimable in this small cohort, precluding meaningful interpretation of a hazard ratio for CaMKK2 expression. Therefore, the current data do not establish CaMKK2 as an independent prognostic marker but rather support its association with high-risk CLL biology in an exploratory setting. In a publicly available independent dataset (GSE22762; Herold et al. [39]), reanalysis using an equal-size median split, consistent with the Duke cohort, showed a directionally similar but non-significant trend toward inferior overall survival among CaMKK2-high cases (log-rank *p* = 0.07585; Cox *p* = 0.0828; 95% CI, 0.91–5.05; SurvExpress [38]; Figure 1D). Finally, in a multivariable linear model evaluating clinical correlates of CaMKK2 expression, including IGHV status and Rai stage, IGHV-unmutated status remained the dominant correlate of higher *CaMKK2* expression (β = 0.496, 95% CI 0.310–0.682, *p* = 1.68 × 10^−7^), whereas Rai stage was not independently associated within this dataset (*p* = 0.307; Supplementary Table S4). This model evaluates predictors of CaMKK2 expression and should not be interpreted as a multivariable survival model testing independent prognostic value. Collectively, these data show that CaMKK2 expression is associated with IGHV-unmutated/high-risk CLL biology and exploratory differences in time to treatment and overall survival. Because clinical variables, including Rai stage, were not uniformly distributed across groups and because multivariable time-to-event modeling was not feasible in this cohort, these analyses do not establish independent prognostic value.

**Figure 2:**
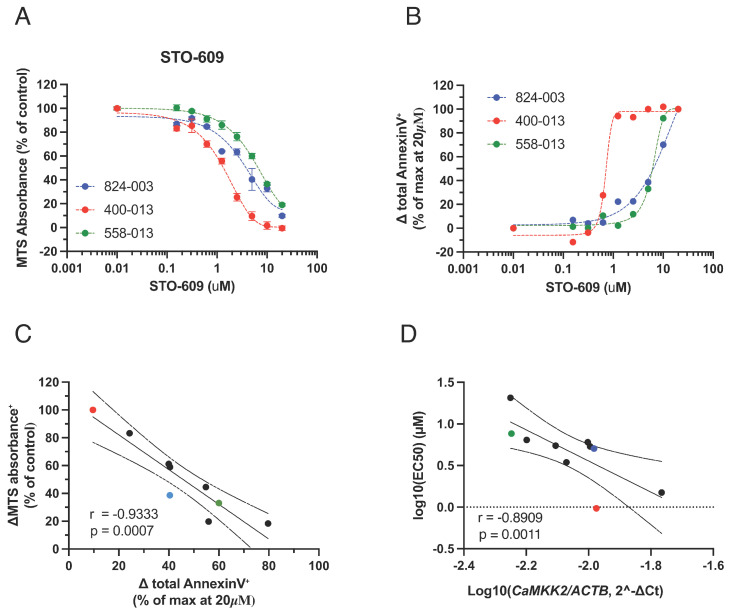
Pharmacological CaMKK2 inhibition is associated with reduced viability and induced cell death in primary CLL cells. Primary CD19^+^ purified CLL cells were isolated from fresh PBMC (n = 9 independent patient samples), and treated with increasing concentrations of STO-609, a pharmacological inhibitor of CaMKK2. CLL viability was assessed using complementary assays of metabolic activity and cell death. (**A**) Representative MTS dose–response curves from three CLL samples (IDs 824-003, 400-013, and 558-013); absorbance is expressed as percentage of vehicle control. (**B**) Representative flow-cytometry dose–response curves from the same samples, reporting total Annexin V^+^ cells (PI^−^ and PI^+^ populations). (**C**) Relationship between metabolic inhibition and cell death at 5 µM STO-609, plotting MTS (% of control) versus Δ total Annexin V^+^ (treated − vehicle), normalized to the maximal response observed at 20 µM for each sample (*n* = 9). (**D**) Association between STO-609 potency and *CaMKK2* expression across primary CLL samples (*n* = 10), plotting MTS-derived EC50 values versus *CaMKK2* expression measured by qRT-PCR and normalized to *ACTB*. Associations were assessed using Spearman rank correlation; regression lines are shown for visualization (black lines). Dots represent individual patient samples, with color coding (red, blue, and green) matching that used for the corresponding panels (**A**,**B**).

Pharmacologic CaMKK2 inhibition with STO-609 was associated with reduced primary CLL viability in a dose-dependent manner. To explore whether pharmacological inhibition of CaMKK2 alters primary CLL viability in vitro, purified primary CD19^+^ CLL cells from nine independent patient samples (clinically annotated in Supplementary Table S2) were treated with increasing concentrations of the CaMKK2 inhibitor STO-609 and evaluated using orthogonal assays of metabolic activity (MTS) and cell death (Annexin V/PI) (Figure 2). Representative dose–response curves from three samples (IDs 824-003, 400-013, and 558-013) demonstrated a concentration-dependent reduction in MTS signal accompanied by an increase in total Annexin V^+^ events (including PI^−^ and PI^+^ populations) (Figure 2A,B). Across the nine samples, STO-609 sensitivity varied (MTS-derived EC50 range of ~0.97–7.67 µM), providing clinically annotated context for inter-sample heterogeneity in response. To distinguish STO-609-induced cytotoxicity from isolated metabolic suppression, we compared MTS response and Annexin V response at a fixed dose. At 5 µM STO-609, MTS viability (% of vehicle control) inversely correlated with the increase in total Annexin V positivity (treated-vehicle; normalized to each sample’s maximal response at 20 µM) (Spearman r = −0.9333, *p* = 0.0007; Figure 2C). Consistent with pathway involvement, but not definitive of on-target activity, STO-609 potency correlated with CaMKK2 expression measured by qRT-PCR (*CaMKK2*/*ACTB*), with higher *CaMKK2* associated with greater sensitivity (Spearman r = −0.8909, *p* = 0.0011; Figure 2D). The observed correlation between CaMKK2 expression and inhibitor sensitivity is consistent with pathway involvement but does not establish direct on-target causality. Supporting a direct pro-apoptotic effect, supplementary analyses showed that STO-609 increased Annexin V positivity across samples independently of baseline apoptosis levels, with no significant association between basal apoptosis and treatment-induced cell death (Figure S2). While STO-609 treatment reduced CLL viability and induced apoptosis, these data should be interpreted with consideration of the known limitations of pharmacologic inhibitors, including potential off-target effects. To further assess whether the observed effects were specific to STO-609, two additional structurally distinct CaMKK2 inhibitors, SGC-CaMKK2-1 [36] and CC-8977 [35], were evaluated in CLL primary cells from an independent cohort of patients that was distinct from the cohort analyzed in Figure 2, including one with documented multi-drug-resistant disease (Supplementary Table S5). Both compounds reduced CLL survival in MTS assay, demonstrating activity broadly comparable to STO-609, although potency varied across samples. Notably, CC-8977 exhibited higher EC50 in the multi-drug-resistant sample. Use of multiple structurally distinct CaMKK2 inhibitors provides partial orthogonal pharmacologic support; however, these approaches do not substitute for genetic or biochemical target-engagement validation.

**Figure 3:**
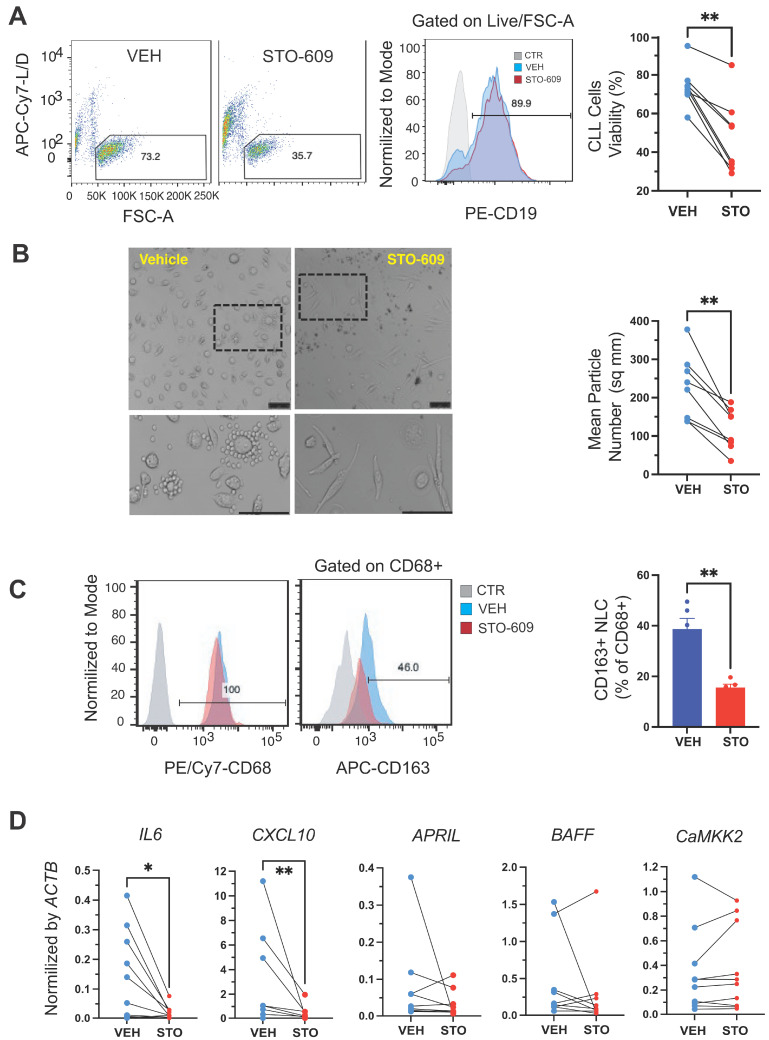
CaMKK2 inhibition during PBMC culture reduces leukemia cell viability and nurse-like cells (NLC) generation. Fresh isolated PBMC from CLL patients were cultured at high density for 14 days in the presence of vehicle (Veh; DMSO) or the CaMKK2 inhibitor STO-609 (5 µM). Non-adherent and adherent fractions were analyzed separately. (**A**) Non-adherent cells were stained for CD19 and viability markers, and CD19^+^ CLL cell viability was quantified by flow cytometry. Representative plots are displayed on the left. Paired data from individual patients are shown on the right. (**B**) Adherent cells were visualized by bright-field microscopy after extensive washing. (Left) Representative images are shown; scale bar = 75 µm. Dashed boxes indicate regions shown at higher magnification in the panel below. Images were analyzed using ImageJ, and the mean number of adherent particles per mm^2^ is quantified (right). (**C**) Adherent cells were detached and analyzed by flow cytometry for macrophage markers. Representative histograms of CD68 and CD163 expression are shown. The right panel quantifies the percentage of CD163^+^ cells within the CD68^+^ population. (**D**) RNA isolated from adherent cells was analyzed by qRT-PCR to quantify expression of genes of interest normalized to *ACTB* and expressed as 2^−ΔCt^. Each line represents paired measurements from an individual patient. Statistical significance was assessed using paired two-tailed tests; *, and ** indicate *p* < 0.05, *p* < 0.01, respectively.

CaMKK2 inhibition during PBMC culture reduces CLL cell viability and alters NLC yield and phenotype. CLL persistence is supported by both cell-autonomous survival programs within the leukemia cell and cell-extrinsic cues provided by the myeloid microenvironment. NLCs provide an established ex vivo model of this protective niche [6], while *CaMKK2* is highly expressed in pro-tumoral macrophage states [23,25,40], raising the possibility that pharmacological inhibition of CaMKK2 may alter the CLL-NLC cross-functional axis that sustains leukemic survival. To test this hypothesis, PBMCs from CLL patients were cultured at high density for 14 days in the presence of vehicle or STO-609 (5 µM) to generate adherent NLC (Figure 3; annotated in Supplementary Table S2). Non-adherent and adherent cells were recovered as described in the Material and Methods section and analyzed for yield, phenotype, and gene expression. A representative staining of PBMC-derived CLL cells at day 0 is shown in Figure S3. Compared with vehicle, STO-609 treatment significantly reduced the percentage of viable CD19^+^ CLL cells recovered in the non-adherent fraction across paired patient samples (Figure 3A). Notably, primary CLL cells exhibit a well-recognized tendency to undergo spontaneous apoptosis in vitro in the absence of microenvironmental support [6,32]. Within this context, the additional reduction in CLL viability observed with CaMKK2 inhibition suggests that CaMKK2 contributes to survival mechanisms that operate beyond basal cell death. This effect is therefore consistent with a modulatory role for CaMKK2 in sustaining CLL viability, acting in conjunction with microenvironment-derived signals rather than as a sole determinant of cell survival. Bright-field imaging after extensive washing of well plates showed reduced adherent cell yield with STO-609, which was confirmed by ImageJ-based particle quantification (Figure 3B). Phenotypic analysis of detached adherent cells demonstrated a decrease in the proportion of CD163^+^ cells within the CD68^+^ macrophage compartment, consistent with impaired acquisition of an NLC-like phenotype (Figure 3C and Figure S4). Thereby, the effect of STO-609 treatment on CLL likely reflects a combination of reduced NLC abundance and altered macrophage phenotype under STO-609 treatment conditions. At the transcript level, *CaMKK2* was detectable in CLL and, in paired samples, was expressed at higher levels in NLC than in autologous CLL cells (Supplementary Figure S5). STO-609 treatment altered expression of macrophage-associated inflammatory mediators (*IL6* and *CXCL10*) [41,42,43,44], which are positively associated with leukemia cell survival and disease progression, whereas *CaMKK2*, *BAFF*, and *APRIL* mRNA levels in adherent NLC were not significantly changed (Figure 3D). Collectively, these data show that CaMKK2 inhibition during PBMC culture is associated with reduced CLL cell persistence and altered NLC characteristic within this experimental system.

**Figure 4:**
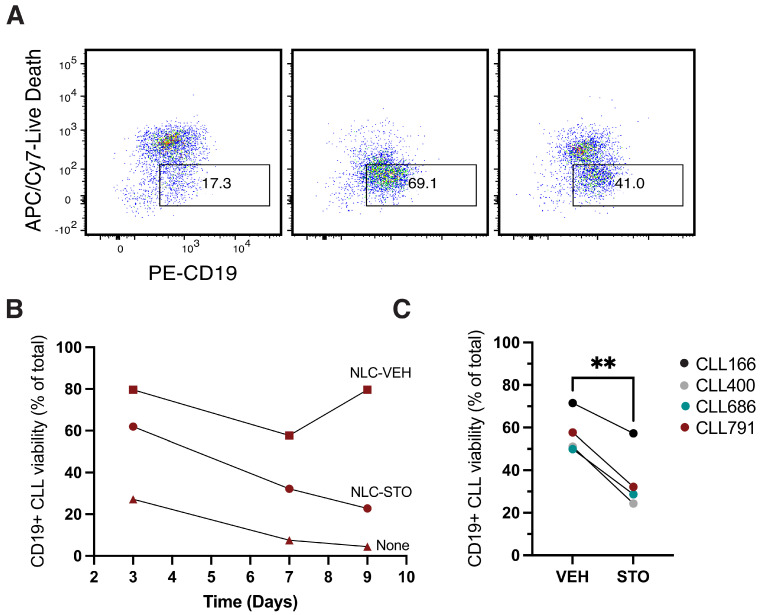
Pharmacological inhibition of CaMKK2 during PBMC culture impairs NLC-mediated support of CLL cell survival. Fresh isolated PBMCs from CLL patients were cultured at high density for 14 days with vehicle or STO-609 (5 µM) to generate adherent NLC. CD19^+^ CLL cells were purified from vehicle cultures, while NLC from vehicle- or STO-609-treated cultures were detached and replated. CLL cells were cultured alone (“None”) or co-cultured with NLC generated under vehicle or STO-609 conditions. (**A**) Representative flow-cytometry plots of viable CD19^+^ CLL cells at day 7 from a representative patient. (**B**) Quantification of live CD19^+^ CLL cells for CLL791 at the indicated time points. Each point represents the mean of technical duplicates from a representative patient. (**C**) Summary of live CD19^+^ CLL cell percentages from four independent CLL patients. All samples were collected at day 7, except for CLL686, which was collected on day 5. Each point represents one independent CLL patient (biological replicate), plotted as the mean of technical triplicates. Paired comparisons were performed using a two-tailed paired *t* test. The distribution of within-sample differences passed the Shapiro–Wilk normality test. ** indicates *p* < 0.01.

STO-609 impairs NLC-mediated support of CLL cell survival. Building on the association between CaMKK2 expression and high-risk CLL biology (Figure 1; Supplementary Table S1), and on the observation that pharmacologic CaMKK2 inhibition was associated with reduced primary CLL viability (Figure 2), we next evaluated whether NLC generated in the presence of STO-609 showed reduced capacity to support CLL survival. PBMCs from CLL patients were cultured at high density for 14 days with vehicle or STO-609 (5 µM) to generate adherent NLC, after which NLC from vehicle- or STO-609-treated cultures were detached, replated, and used as feeder cells for autologous CD19^+^ CLL cells purified from the vehicle condition (Figure 4 and annotated in Supplementary Table S2). As expected, CLL cells cultured alone (“None”) showed progressive loss of viability over time, whereas co-culture with vehicle-derived NLC (NLC-Veh) increased recovery of live non-adherent CD19^+^ cells at days 3 and 7, confirming the protective function of NLC in this system (Figure 4A,B). In contrast, NLC generated in the presence of STO-609 (NLC-STO) exhibited a markedly reduced capacity to sustain CLL viability, resulting in significantly fewer live CD19^+^ cells compared with NLC-Veh at both time points (Figure 4A,B). These effects were reproducible across four independent CLL patient samples (Figure 4C). Paired comparisons were performed using a two-tailed paired *t*-test. The distribution of within-sample differences passed the Shapiro–Wilk normality test; however, the small number of biological replicates limits the robustness of this assessment. Interpretation of these results is further limited by the inclusion of one sample (CLL686) collected at day 5, while all other post-treatment samples were collected at day 7. NLCs were extensively washed prior to co-culture to minimize inhibitor carryover, making a direct effect of STO-609 on CLL cells unlikely. In addition, an equal number of NLCs generated under each condition were used in secondary co-culture experiments, reducing the contribution of feeder cell abundance and supporting a role for qualitative changes in macrophage function. The observed reduction in CLL support therefore reflects intrinsic alterations in NLC function. Collectively, these data indicate that pharmacological inhibition of CaMKK2 is associated with reduced NLC-mediated support of CLL cells in ex vivo models, consistent with both qualitative and quantitative changes in macrophage function.

**Figure 5:**
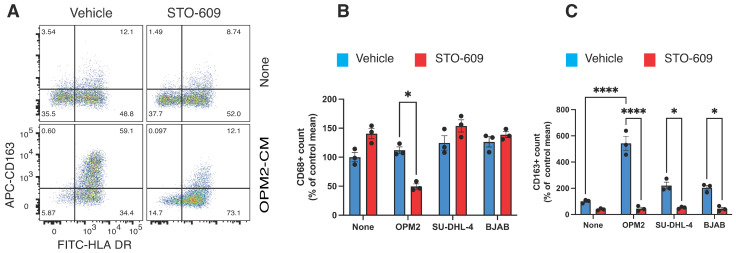
Tumor-derived signals induce an immunosuppressive NLC phenotype that is attenuated by pharmacological CaMKK2 inhibition. Monocyte-derived macrophages from healthy donors (n = 3) were cultured in the absence (“None”) or presence of tumor-conditioned media, with vehicle or STO-609 (5 µM), and analyzed by flow cytometry. (**A**) Representative flow-cytometry plots showing HLA-DR and CD163 expression on CD14^+^CD68^+^ macrophages cultured with or without OPM2-conditioned medium (OPM2-CM). (**B**,**C**) Quantification of total CD68^+^ macrophages and the CD163^+^ subset following exposure to conditioned media from OPM2, SU-DHL-4, or BJAB cells. CaMKK2 inhibition limits acquisition of a CD163^+^ immunosuppressive phenotype induced by tumor-derived factors. Data are presented as mean ± SEM from three independent biological donors (n = 3; black dots). For each donor, technical triplicates were averaged. Paired comparisons across conditions were analyzed using two-way repeated-measures ANOVA with donor as the matched factor, followed by post hoc multiple-comparisons testing. Given the limited number of biological donors (n = 3), findings should be interpreted with caution; significance is indicated in the figure. * and **** indicate *p* < 0.05 and *p* < 0.0001, respectively.

Tumor-conditioned media drives an immunosuppressive macrophage phenotype that is attenuated by pharmacological CaMKK2 inhibition. To test whether CaMKK2 contributes to tumor-driven programming of macrophage phenotypes relevant to the CLL microenvironment, we generated monocyte-derived macrophages (MDMs) from three independent healthy donor leukocytes and cultured them either in control conditions (“None”) or in the presence of tumor-conditioned media (TCMs) from the B-cell malignancy cell lines OPM2, SU-DHL-4, or BJAB, with vehicle or STO-609 (5 µM) (Figure 5). Flow-cytometric analysis based on absolute cell yield, normalized to basal conditions and expressed as percentage of control mean, showed that exposure to tumor-conditioned media (TCMs) increased the proportion of CD163^+^ cells within the CD68^+^ macrophage compartment (Figure 5A–C), consistent with enrichment of an NLC-like phenotype (Figure 5A). Analysis of total CD68^+^ macrophage yield indicated no consistent changes across conditions when expressed relative to basal levels (Figure 5B), supporting the notion that TCMs primarily influence macrophage polarization rather than overall macrophage accumulation. A reduction in CD68^+^ yield was observed upon STO-609 treatment in the OPM2 condition, but this effect was not consistent across all TCM conditions (Figure 5B).

In contrast, TCMs markedly increased CD163^+^ macrophage yield across all conditioned media sources (OPM2, SU-DHL-4, BJAB), with values ranging approximately from 200% to 800% of basal levels (Figure 5C). Despite this consistent trend, statistical significance was reached only in the OPM2 condition, whereas variability among biological replicates and the influence of high-value measurements in other conditions likely limited statistical power and affected the two-way ANOVA results. Notably, pharmacologic inhibition of CaMKK2 with STO-609 significantly reduced the CD163^+^ macrophage yield across all tested conditions (Figure 5C). Together with the patient-derived NLC data (Figure 3 and Figure 4) and our prior studies of CaMKK2 genetic ablation in macrophages exposed to tumor-conditioned media [23], these findings support an association between CaMKK2 activity and macrophage responses to tumor-derived signals. However, given the limited number of biological replicates, the resulting constraints on statistical power, and lack of complementary genetic evidence in human macrophage, these observations should be interpreted with caution, and causality cannot be definitively established based on pharmacological inhibition alone.

## 4. Discussion

In this study, we report that CaMKK2 expression is associated with high-risk CLL biology and that pharmacologic inhibition modulates leukemic and macrophage-associated phenotypes in ex vivo systems. These findings are exploratory and provide a framework for further investigation into CaMKK2 in CLL.

At the tumor cell level, elevated CaMKK2 expression in purified CD19^+^ CLL cells was associated with shorter time to treatment, inferior overall survival, and enriched in IGHV-unmutated disease. However, these clinical associations are derived from a small and clinically heterogeneous cohort and may be influenced by imbalances in established prognostic variables, including disease stage. The prognostic analyses presented here are exploratory and are based on dichotomized CaMKK2 expression. Because the limited cohort size and small number of outcome events precluded reliable estimation of continuous Cox proportional hazards models, independent prognostic value could not be assessed in this cohort. Further evaluation of CaMKK2 as a continuous variable, including formal Cox regression modeling in larger cohorts, will be necessary to more rigorously define its prognostic utility. Notably, CaMKK2 expression strongly co-segregates with IGHV-unmutated disease, and multivariable analyses have identified IGHV status as the primary determinant of CaMKK2 levels. Accordingly, the prognostic associations observed here should be interpreted in the context of known risk features, and do not support CaMKK2 as an independent prognostic factor. Rather, these findings position CaMKK2 as a biologically relevant marker of high-risk disease programs, linking calcium signaling to leukemic cell fitness. Given that IGHV-unmutated CLL exhibits heightened and sustained BCR signaling and enhanced calcium flux [3,4], these observations suggest that CaMKK2 marks a calcium-dependent, metabolically resilient leukemic state.

Mechanistically, prior studies have shown that store-operated calcium entry (SOCE) via ORAI1/STIM supports intracellular signaling pathways involved in leukemia survival [12,45]. CaMKK2 has been reported to function downstream of Ca^2+^/calmodulin to activate AMPK metabolic stress-adaptation programs [15], and has been proposed to link calcium entry with mitochondrial fitness [24,46]. In this context, the observed effects of CaMKK2 inhibition on leukemic and macrophage phenotypes in vitro suggest that this pathway may influence both tumor-intrinsic and microenvironmental processes. However, the interpretation of our pharmacologic data requires caution because AMPK can also be regulated through CaMKK2-independent mechanisms, including LKB1-dependent signaling, and STO-609 or related inhibitors may have pathway-overlapping or off-target effects. Further studies, including genetic manipulation of CaMKK2 expression, will be required to provide direct evidence on the role of CaMKK2 in this CLL pro-survival circuit. Notably, recent structural analyses of aggressive stereotyped CLL subset 1 indicated that autonomous BCR–homotypic signaling is not universally conserved, indicating that alternative mechanisms may contribute to sustained intracellular signaling and leukemic fitness [47]. In this context, CaMKK2-dependent signaling may represent a parallel or compensatory pathway. Compared to BTK inhibitors, which target proximal BCR signaling nodes, CaMKK2 blockade may affect downstream calcium-dependent metabolic programs, although this distinction is inferred from prior studies rather than directly demonstrated here. Importantly, direct biochemical evidence of CaMKK2 pathway engagement in primary CLL cells, including modulation of canonical downstream targets such as AMPK or AKT phosphorylation, was not assessed in this study.

CLL progression depends on intrinsic biological features of leukemia cells as well as niche-mediated support [48,49]. Within lymph node and marrow niches, NLCs and other myeloid populations provide cytokines and direct contact signals that reinforce BCR-associated pathways, sustain metabolic adaptation, and limit apoptosis. These inputs can attenuate responses to targeted agents and contribute to minimal residual disease. Our data demonstrate that pharmacological inhibition of CaMKK2 alters this protective axis in ex vivo models. STO-609 reduced the yield of CD163^+^ macrophage, altered inflammatory mediator expression, and impaired macrophage-mediated survival of autologous CLL cells. Overall, our findings support a key contribution of qualitative changes in macrophage function, although reduced NLC yield may further contribute and act synergistically. These data align with prior work showing that myeloid CaMKK2 is required to link tumor-derived signal with AMPK activation and transcriptional programs regulating M2-like/pro-tumoral functions and, in turn, tumor progression in breast cancer and lymphoma models [23,24]. Recent evidence further indicate that in murine macrophage CaMKK2 couples the matrix-mediated mechanosensory signaling with AMPK and AKT activation, thereby further expanding this framework [25]. Lymph node remodeling and altered extracellular matrix composition are recognized features of aggressive CLL, and CaMKK2 may function at the convergence of calcium signaling, metabolic adaptation, and biomechanical cues within these niches [5,7,8,9,10]. Thus, CaMKK2 may be associated with biochemical and structural survival inputs across both leukemic and myeloid compartments.

The observed leukemic and macrophage-associated phenotypes may have biological implications for microenvironmental interactions in CLL. While BTK and BCL2 inhibitors disrupt key survival pathways, microenvironmental protection remains a barrier to durable disease control. Increasing evidence indicates that macrophage-rich, metabolically competitive, and structural remodeled niches contribute to the comparatively limited durability of CAR-T cell therapy in CLL relative to other B-cell malignancies [50,51,52]. Notably, recent evidence suggests that pharmacologic CaMKK2 inhibition can synergize with CAR-T therapy in xenograft models [53]. Immunosuppressive macrophages, cytokine gradients, and matrix-associated constraints can impair T-cell fitness and persistence. By simultaneously reducing leukemic metabolic resilience and attenuating macrophage-mediated support, pharmacological inhibition of CaMKK2 may recalibrate this hostile microenvironment.

Recent studies indicate that CLL-cell fitness is regulated by additional microenvironmental mechanisms beyond those examined here. Inhibitory immune checkpoint receptors, including PD-1, PD-L1, LAG-3, and related pathways, may influence CLL biology through effects on immune-cell dysfunction, leukemic-cell activation, and bidirectional interactions within the tumor microenvironment [54]. In parallel, CLL cells engage a complex stromal compartment that includes not only nurse-like cells but also mesenchymal stromal cells, fibroblast-like cells, and fibrocyte-like populations, which provide adhesion-dependent and soluble pro-survival signals [7]. Recent work also indicates that stromal fibroblasts can be functionally programmed to support leukemic progression, further emphasizing the complexity of the protective CLL niche [55]. Although our study focused primarily on purified CLL cells and NLC-mediated support, the finding that CaMKK2 inhibition impairs both CLL cell viability and NLC pro-survival activity suggests that CaMKK2-dependent signaling may intersect with broader microenvironmental survival programs. Future studies using more comprehensive human-relevant models will be required to define how inhibitory receptor signaling, stromal-cell interactions, and CaMKK2-dependent pathways integrate to regulate CLL survival and therapeutic response.

Several study design limitations warrant consideration. The clinical cohort is modest in size and was intentionally balanced for IGHV mutation status rather than fully matched across clinical variables; as a result, imbalances in factors such as Rai stage and treatment history may confound outcome analyses. This study is exploratory in nature, is not powered for multivariate prognostic modeling, and is not designed to establish independent prognostic value or direct CaMKK2-dependent causality. A key limitation of this study is the reliance on pharmacologic inhibition to infer CaMKK2 function. STO-609 has been widely used as selective CaMKK2 inhibitor in pre-clinical models [23,24,56,57,58,59,60], and additional compounds such as SGC-CaMKK2-1 and CC-8977 have demonstrated activity and selectivity across various systems [35,36,61]. While multiple inhibitors were used, off-target effects cannot be excluded. Furthermore, direct target engagement and downstream pathway modulation (e.g., p-AMPK, p-ACC) were not evaluated, and genetic validation approaches were not performed. In addition, STO-609 has reported off-target activity against several kinases, including direct effects on AMPK-related signaling at concentrations overlapping those used in this study. Consequently, the observed phenotypes cannot be attributed exclusively to inhibition of the CaMKK2-AMPK axis, further underscoring the need for direct target-engagement assays and complementary genetic validation. An additional limitation is that CaMKK2 expression was used as a proxy for pathway involvement, although kinase abundance does not necessarily reflect enzymatic activity. While increased CaMKK2 levels may suggest biological relevance, direct assessment of kinase activation and downstream signaling was not performed. Accordingly, the associations reported here should be interpreted in the context of CaMKK2 expression rather than confirmed functional activity. The ex vivo systems used in this study, including PBMC-derived NLC models and tumor-conditioned media assays, represent simplified approximations of the CLL microenvironment and do not fully recapitulate in vivo macrophage ontogeny. CaMKK2 expression may vary during culture due to shifts in PBMC composition and/or differentiation of monocytes into macrophages/NLC, and prolonged culture conditions may influence pathway activity. While our data suggest that CaMKK2 inhibition affects both leukemic cells and macrophage-mediated support, including potential stage-dependent effects during differentiation, these dynamics were not directly resolved. Accordingly, the findings should be interpreted as reflecting CaMKK2-dependent interactions under defined experimental conditions, and further time-resolved and in vivo studies will be required to more fully define its role across macrophage and microenvironmental biology. In addition, our microenvironmental experiments rely on ex vivo PBMC-derived models which capture key aspects of NLC biology but do not fully reproduce the spatial and cellular complexity of lymph node niches. Finally, while the cohort was intentionally balanced by IGHV status, validation in larger, independently annotated datasets with multivariable modeling will be necessary to confirm the prognostic utility of CaMKK2 in CLL. Overall, these limitations underscore the need for mechanistic and clinically annotated studies to define the functional and translational significance of CaMKK2 in CLL.

## 5. Conclusions

In summary, CaMKK2 expression is associated with IGHV-unmutated, high-risk CLL. Pharmacologic inhibition of CaMKK2 was associated with reduced leukemic cell viability and altered macrophage-associated phenotypes in ex vivo systems. These findings suggest that CaMKK2 may reflect underlying disease biology and represent a candidate pathway influencing leukemic and microenvironmental interactions. However, these data do not establish independent prognostic value, direct on-target causality, or therapeutic applicability. Further larger studies will be required to define the biological and clinical role of CaMKK2 in CLL.

## Data Availability

The datasets generated and/or analyzed during the current study are available from the corresponding author upon reasonable request. Publicly available transcriptomic data used for validation were obtained from the Gene Expression Omnibus (GEO) under accession number GSE22762.

## Supplementary Materials

The following supporting information can be downloaded at: https://www.mdpi.com/article/10.3390/cells15141294/s1, Supplementary Table S1. Demographic and clinical summary of CLL patient samples used for association studies (Figure 1). Supplementary Table S2. Demographic and clinical summary of CLL patient samples used for in vitro studies. Supplementary Table S3. List of primers used for qRT-PCR gene expression analysis. Supplementary Table S4. Multivariable linear regression of CaMKK2 expression with IGHV mutation status and Rai stage (Duke CLL cohort). Supplementary Table S5. Figure S1. Canonical CLL prognostic markers CD38 and ZAP-70 are increased in IGHV unmutated cases. Flow cytometry was performed on peripheral blood CD19^+^ CLL cells to quantify ZAP-70 (A) and CD38 (B) expression. Samples were stratified by IGHV mutation status (M-CLL vs. U-CLL). Each symbol represents one patient; horizontal bars indicate mean ± SEM. Group differences were assessed using the Mann-Whitney test; significance is indicated by asterisks. Figure S2. CaMKK2 inhibition increases apoptosis in primary CLL cells independently of basal apoptosis levels. Primary CLL cells isolated from fresh PBMC samples were treated with STO-609 or vehicle control. (A) Flow-cytometry analysis of Annexin V positivity showed increased apoptosis after treatment with 5 μM STO-609 compared with vehicle control. Each paired point represents an independent patient sample. (B) The STO-609-induced increase in apoptosis, calculated as ΔAnnexin V positivity relative to matched vehicle-treated cells, was plotted against basal apoptosis levels measured in vehicle-treated cells. No significant association was observed between basal apoptosis and the magnitude of STO-609-induced apoptosis, suggesting that the pro-apoptotic effect of CaMKK2 inhibition is not simply explained by higher pre-existing cell death. Associations were assessed by Spearman rank correlation; regression line is shown for visualization. * *p* < 0.05. Figure S3. Gating strategy and baseline phenotype of a representative PBMC-CLL sample at day 0 of long-term culture. Representative flow-cytometry plots of a PBMCCLL sample at day 0 of long-term culture. The left panel shows the initial cell gate based on forward and side scatter properties. The middle panel shows identification of viable cells using live/dead staining, based on the left panel gate. The right panel shows CD19 expression within the live-cell gate. Numbers indicate the percentage of events within the indicated gates. The CD19 gate was set using the fluorescence-minus-one (FMO) control. Figure S4. Flow cytometry gating strategy for identification of CD163^+^ macrophages. Representative flow cytometry plots illustrating the sequential gating strategy used to identify CD163^+^ macrophages derived from peripheral blood mononuclear cell (PBMC) of CLL and healthy donors. Detached adherent cells were first gated based on forward and side scatter (FSC-A/SSC-A) parameters to exclude debris and select the viable cell population. Doublets were excluded using FSC-area versus FSC-height gating (FSC-A/FSC-H). The macrophage population was then defined based on CD68 expression. Within this gated CD68^+^ compartment, CD163 expression was assessed to quantify the CD163^+^ macrophage subset. Histograms show representative CD163 staining with corresponding gating thresholds. Percentage values indicate the proportion of CD163^+^ cells within the CD68^+^ macrophage population. Gates were defined using fluorescence minus one (FMO) controls where applicable. Figure S5. CaMKK2 expression in CLL and corresponding autologous NLC. CaMKK2 expression in CLL cells and paired autologous NLC. PBMCs from CLL patients were cultured at high density for 14 days to generate a non-adherent CD19^+^ CLL fraction and an adherent NLC-enriched fraction. Left: CaMKK2 mRNA expression (CaMKK2/ACTB) measured by qRT-PCR in purified CD19^+^ CLL cells (CLL) and matched autologous adherent NLC; each line connects paired measurements from the same patient. Statistical significance was assessed using a two-tailed paired Wilcoxon signed-rank test; *p* < 0.05. Right: Estimation plot showing CaMKK2 expression in CLL and NLC, and the paired difference (NLC-CLL), expressed as a percentage of the mean CaMKK2/ACTB value in the CLL group; points represent individual patients and error bars indicate the mean difference with 95% confidence interval.

## References

[1] Hallek M. (2025). Chronic Lymphocytic Leukemia: 2025 Update on the Epidemiology, Pathogenesis, Diagnosis, and Therapy. Am. J. Hematol..

[2] Shadman M. (2023). Diagnosis and Treatment of Chronic Lymphocytic Leukemia: A Review. JAMA J. Am. Med. Assoc..

[3] Dühren-von Minden M., Übelhart R., Schneider D., Wossning T., Bach M.P., Buchner M., Hofmann D., Surova E., Follo M., Köhler F. (2012). Chronic lymphocytic leukaemia is driven by antigen-independent cell-autonomous signalling. Nature.

[4] Iatrou A., Agathangelidis A., Bordini J., Stamatopoulos K., Ghia P. (2025). Autonomous B-cell Receptor Signaling in Chronic Lymphocytic Leukemia. Hematol. Oncol. Clin. N. Am..

[5] Fiorcari S., Maffei R., Atene C.G., Potenza L., Luppi M., Marasca R. (2021). Nurse-Like Cells and Chronic Lymphocytic Leukemia B Cells: A Mutualistic Crosstalk inside Tissue Microenvironments. Cells.

[6] Burger J.A., Tsukada N., Burger M., Zvaifler N.J., Dell’Aquila M., Kipps T.J. (2000). Blood-derived nurse-like cells protect chronic lymphocytic leukemia B cells from spontaneous apoptosis through stromal cell–derived factor-1. Blood.

[7] Vom Stein A.F., Hallek M., Nguyen P.H. (2024). Role of the tumor microenvironment in CLL pathogenesis. Semin. Hematol..

[8] Panayiotidis P., Jones D., Ganeshaguru K., Foroni L., Hoffbrand A.V. (1996). Human bone marrow stromal cells prevent apoptosis and support the survival of chronic lymphocytic leukaemia cells in vitro. Br. J. Haematol..

[9] De Oliveira T.D., Vom Stein A., Rebollido-Rios R., Lobastova L., Lettau M., Janssen O., Wagle P., Nguyen P.-H., Hallek M., Hansen H.P. (2023). Stromal cells support the survival of human primary chronic lymphocytic leukemia (CLL) cells through Lyn-driven extracellular vesicles. Front. Med..

[10] Dubois N., Crompot E., Meuleman N., Bron D., Lagneaux L., Stamatopoulos B. (2020). Importance of Crosstalk Between Chronic Lymphocytic Leukemia Cells and the Stromal Microenvironment: Direct Contact, Soluble Factors, and Extracellular Vesicles. Front. Oncol..

[11] Berditchevski F., Fennell E., Murray P.G. (2021). Calcium-dependent signalling in B-cell lymphomas. Oncogene.

[12] Scaviner J., Bagacean C., Christian B., Renaudineau Y., Mignen O., Abdoul-Azize S. (2024). Blocking Orai1 constitutive activity inhibits B-cell cancer migration and synergistically acts with drugs to reduce B-CLL cell survival. Eur. J. Pharmacol..

[13] Sana I., Mantione M.E., Angelillo P., Muzio M. (2021). Role of NFAT in Chronic Lymphocytic Leukemia and Other B-Cell Malignancies. Front. Oncol..

[14] Arthur R., Wathen A., Lemm E.A., Stevenson F.K., Forconi F., Linley A.J., Steele A.J., Packham G., Valle-Argos B. (2022). BTK-independent regulation of calcium signalling downstream of the B-cell receptor in malignant B-cells. Cell. Signal..

[15] Racioppi L., Means A.R. (2012). Calcium/calmodulin-dependent protein kinase kinase 2: Roles in signaling and pathophysiology. J. Biol. Chem..

[16] Pulliam T.L., Goli P., Awad D., Lin C., Wilkenfeld S.R., Frigo D.E. (2022). Regulation and role of CAMKK2 in prostate cancer. Nat. Rev. Urol..

[17] Mukherjee D., Previs R.A., Haines C., Al Abo M., Juras P.K., Strickland K.C., Chakraborty B., Artham S., Whitaker R.S., Hebert K. (2023). Targeting CaMKK2 Inhibits Actin Cytoskeletal Assembly to Suppress Cancer Metastasis. Cancer Res..

[18] Wu Y., Jia C., Liu W., Zhan W., Chen Y., Lu J., Bao Y., Wang S., Yu C., Zheng L. (2024). Sodium citrate targeting Ca. J. Adv. Res..

[19] Gocher A.M., Azabdaftari G., Euscher L.M., Dai S., Karacosta L.G., Franke T.F., Edelman A.M. (2017). Akt Activation by Ca^2+^/calmodulin-dependent protein Kinase Kinase 2 (CaMKK2) in Ovarian Cancer Cells. J. Biol. Chem..

[20] Najar M.A., Modi P.K., Ramesh P., Sidransky D., Gowda H., Prasad T.S.K., Chatterjee A. (2021). Molecular Profiling Associated with Calcium/Calmodulin-Dependent Protein Kinase Kinase 2 (CAMKK2)-Mediated Carcinogenesis in Gastric Cancer. J. Proteome Res..

[21] Subbannayya Y., Syed N., Barbhuiya M.A., Raja R., Marimuthu A., Sahasrabuddhe N., Pinto S.M., Manda S.S., Renuse S., Manju H.C. (2015). Calcium calmodulin dependent kinase kinase 2-a novel therapeutic target for gastric adenocarcinoma. Cancer Biol. Ther..

[22] Tomaszewski W.H., Waibl-Polania J., Chakraborty M., Perera J., Ratiu J., Miggelbrink A., McDonnell D.P., Khasraw M., Ashley D.M., Fecci P.E. (2022). Neuronal CaMKK2 promotes immunosuppression and checkpoint blockade resistance in glioblastoma. Nat. Commun..

[23] Racioppi L., Nelson E.R., Huang W., Mukherjee D., Lawrence S.A., Lento W., Masci A.M., Jiao Y., Park S., York B. (2019). CaMKK2 in myeloid cells is a key regulator of the immune-suppressive microenvironment in breast cancer. Nat. Commun..

[24] Huang W., Liu Y., Luz A., Berrong M., Meyer J.N., Zou Y., Swann E., Sundaramoorthy P., Kang Y., Jauhari S. (2021). Calcium/Calmodulin Dependent Protein Kinase Kinase 2 Regulates the Expansion of Tumor-Induced Myeloid-Derived Suppressor Cells. Front. Immunol..

[25] Guan Y., Zhang M., Song J., Negrete M., Adcock T., Kandel R., Racioppi L., Gerecht S. (2025). CaMKK2 Regulates Macrophage Polarization Induced by Matrix Stiffness: Implications for Shaping the Immune Response in Stiffened Tissues. Adv. Sci..

[26] Crombie J., Davids M.S. (2017). *IGHV* mutational status testing in chronic lymphocytic leukemia. Am. J. Hematol..

[27] Katagiri S., Yonezawa T., Kuyama J., Kanayama Y., Nishida K., Abe T., Tamaki T., Ohnishi M., Tarui S. (1985). Two distinct human myeloma cell lines originating from one patient with myeloma. Int. J. Cancer.

[28] Clements G.B., Klein G., Povey S. (1975). Production by EBV infection of an EBNA-positive subline from an EBNA-negative human lymphoma cell line without detectable EBV DNA. Int. J. Cancer.

[29] Epstein A.L., Kaplan H.S. (1979). Feeder layer and nutritional requirements for the establishment and cloning of human malignant lymphoma cell lines. Cancer Res..

[30] Zarif J.C., Hernandez J.R., Verdone J.E., Campbell S.P., Drake C.G., Pienta K.J. (2016). A phased strategy to differentiate human CD14+monocytes into classically and alternatively activated macrophages and dendritic cells. Biotechniques.

[31] Levesque M.C., Ghosh D.K., Beasley B.E., Chen Y., Volkheimer A.D., O’Loughlin C.W., Gockerman J.P., Moore J.O., Weinberg J.B. (2008). CLL cell apoptosis induced by nitric oxide synthase inhibitors: Correlation with lipid solubility and NOS1 dissociation constant. Leuk. Res..

[32] Levesque M., O’Loughlin C., Weinberg J. (2001). Use of serum-free media to minimize apoptosis of chronic lymphocytic leukemia cells during in vitro culture. Leukemia.

[33] Friedman D.R., Lanasa M.C., Davis P.H., Allgood S.D., Matta K.M., Brander D.M., Chen Y., Davis E.D., Volkheimer A.D., Moore J.O. (2014). Perifosine treatment in chronic lymphocytic leukemia: Results of a phase II clinical trial and in vitro studies. Leuk. Lymphoma.

[34] Tokumitsu H., Inuzuka H., Ishikawa Y., Ikeda M., Saji I., Kobayashi R. (2002). STO-609, a specific inhibitor of the Ca(2+)/calmodulin-dependent protein kinase kinase. J. Biol. Chem..

[35] Chen Y., Whitefield B., Nevius E., Hill M., DelRosario J., Sinitsyna N., Shanmugasundaram V., Mukherjee D., Shi L., Mayne C.G. (2023). Identification of Small Molecule Inhibitors and Ligand Directed Degraders of Calcium/Calmodulin Dependent Protein Kinase Kinase 1 and 2 (CaMKK1/2). J. Med. Chem..

[36] Wells C., Liang Y., Pulliam T.L., Lin C., Awad D., Eduful B., O’byrne S., Hossain M.A., Catta-Preta C.M.C., Ramos P.Z. (2023). SGC-CAMKK2-1: A Chemical Probe for CAMKK2. Cells.

[37] Sattiraju A., Kang S., Giotti B., Chen Z., Marallano V.J., Brusco C., Ramakrishnan A., Shen L., Tsankov A.M., Hambardzumyan D. (2023). Hypoxic niches attract and sequester tumor-associated macrophages and cytotoxic T cells and reprogram them for immunosuppression. Immunity.

[38] Aguirre-Gamboa R., Gomez-Rueda H., Martínez-Ledesma E., Martínez-Torteya A., Chacolla-Huaringa R., Rodriguez-Barrientos A., Tamez-Peña J.G., Treviño V. (2013). SurvExpress: An Online Biomarker Validation Tool and Database for Cancer Gene Expression Data Using Survival Analysis. PLoS ONE.

[39] Herold T., Jurinovic V., Metzeler K.H., Boulesteix A.-L., Bergmann M., Seiler T., Mulaw M., Thoene S., Dufour A., Pasalic Z. (2011). An eight-gene expression signature for the prediction of survival and time to treatment in chronic lymphocytic leukemia. Leukemia.

[40] Racioppi L., Noeldner P.K., Lin F., Arvai S., Means A.R. (2012). Calcium/calmodulin-dependent protein kinase kinase 2 regulates macrophage-mediated inflammatory responses. J. Biol. Chem..

[41] Castellanos S.V., Castellanos M.P.R., Avendaño M.C.G., Asprilla M.C.A., Leal M.S.G., Tirado G. (2025). Impact of the tumor microenvironment on progression and treatment response in lymphoma and chronic lymphocytic leukemia: A systematic review of the literature. Crit. Rev. Oncol. Hematol..

[42] Uziel O., Lipshtein L., Sarsor Z., Beery E., Bogen S., Lahav M., Regev A., Kliminski V., Sharan R., Gervits A. (2024). Chronic Lymphocytic Leukemia (CLL)-Derived Extracellular Vesicles Educate Endothelial Cells to Become IL-6-Producing, CLL-Supportive Cells. Biomedicines.

[43] Mitrović-Ajtić O., Živković E., Subotički T., Diklić M., Đikić D., Vukotić M., Dragojević T., Vuković V., Antić D., Čokić V.P. (2024). Inflammation mediated angiogenesis in chronic lymphocytic leukemia. Ann. Hematol..

[44] Yan X.-J., Dozmorov I., Li W., Yancopoulos S., Sison C., Centola M., Jain P., Allen S.L., Kolitz J.E., Rai K.R. (2011). Identification of outcome-correlated cytokine clusters in chronic lymphocytic leukemia. Blood.

[45] Debant M., Burgos M., Hemon P., Buscaglia P., Fali T., Melayah S., Le Goux N., Vandier C., Potier-Cartereau M., Pers J.-O. (2019). STIM1 at the plasma membrane as a new target in progressive chronic lymphocytic leukemia. J. Immunother. Cancer.

[46] Rabinovitch R.C., Samborska B., Faubert B., Ma E.H., Gravel S.P., Andrzejewski S., Raissi T.C., Pause A., St-Pierre J., Jones R.G. (2017). AMPK Maintains Cellular Metabolic Homeostasis through Regulation of Mitochondrial Reactive Oxygen Species. Cell Rep..

[47] Cocomazzi P.G., Iatrou A., Minici C., Gounari M., Linder A.T., Akpınaroğlu C., Patrone M., Broggini L., Frenquelli M., Sarrigeorgiou I. (2025). Defective cell-autonomous signalling and antigenic polyreactivity of B-cell receptors from chronic lymphocytic leukaemia stereotyped subset 1. Nat. Commun..

[48] Jestrabek H., Kohlhas V., Hallek M., Nguyen P.H. (2024). Impact of leukemia-associated macrophages on the progression and therapy response of chronic lymphocytic leukemia. Leuk. Res..

[49] Dumontet E., Mancini S.J.C., Tarte K. (2021). Bone Marrow Lymphoid Niche Adaptation to Mature B Cell Neoplasms. Front. Immunol..

[50] Beyar-Katz O., Benjamini O., Delgado J., Ruella M., Ram R., Grisariu S., Visentin A., Vandenberghe E., Gentile M., Avigdor A. (2025). CD19 CAR T-Cell Therapy in Richter Transformation: A Multicentre Retrospective Analysis by the European Research Initiative on Chronic Lymphocytic Leukaemia. J. Cell. Mol. Med..

[51] Derigs P., Schubert M.-L., Dreger P., Schmitt A., Yousefian S., Haas S., Röthemeier C., Neuber B., Hückelhoven-Krauss A., Brüggemann M. (2024). Third-generation anti-CD19 CAR T cells for relapsed/refractory chronic lymphocytic leukemia: A phase 1/2 study. Leukemia.

[52] Can I., Cox M.J., Siegler E.L., Sakemura R., Kenderian S.S. (2022). Challenges of chimeric antigen receptor T-cell therapy in chronic lymphocytic leukemia: Lessons learned. Exp. Hematol..

[53] Geiger R., Vogel I., Casagranda A., Cattaneo M., Morosi L., Pecoraro M., Sulheim E., Basso C. (2025). RNF19B confers tumor resistance to CAR T cells. Res. Sq..

[54] Llaó-Cid L., Wong J., Fernandez Botana I., Paul Y., Wierz M., Pilger L.-M., Floerchinger A., Tan C., Gonder S., Pagano G. (2025). Integrative multi-omics reveals a regulatory and exhausted T-cell landscape in CLL and identifies galectin-9 as an immunotherapy target. Nat. Commun..

[55] Vom Stein A.F., Rebollido-Rios R., Lukas A., Koch M., Von Lom A., Reinartz S., Bachurski D., Rose F., Bozek K., Abdallah A.T. (2023). LYN kinase programs stromal fibroblasts to facilitate leukemic survival via regulation of c-JUN and THBS1. Nat. Commun..

[56] York B., Li F., Lin F., Marcelo K.L., Mao J., Dean A., Gonzales N., Gooden D., Maity S., Coarfa C. (2017). Pharmacological inhibition of CaMKK2 with the selective antagonist STO-609 regresses NAFLD. Sci. Rep..

[57] Zhou Y., Wang Y., Wang M., Li B., Xu K., Pan X., Li Z., Wang Q., He W., Pang J. (2025). CAMKK2 restored mitochondrial dynamics homeostasis to alleviate pulmonary fibrosis via AMPK/PGC-1α signaling pathway in lung fibroblasts. Mol. Med..

[58] Chen M., Dong X., Deng H., Ye F., Zhao Y., Cheng J., Dan G., Zhao J., Sai Y., Bian X. (2021). Targeting TRPV1-mediated autophagy attenuates nitrogen mustard-induced dermal toxicity. Signal Transduct. Target. Ther..

[59] Ma Z., Wen D., Wang X., Yang L., Liu T., Liu J., Zhu J., Fang X. (2016). Growth inhibition of human gastric adenocarcinoma cells in vitro by STO-609 is independent of calcium/calmodulin-dependent protein kinase kinase-beta and adenosine monophosphate-activated protein kinase. Am. J. Transl. Res..

[60] EMassie C., Lynch A., Ramos-Montoya A., Boren J., Stark R., Fazli L., Warren A., Scott H., Madhu B., Sharma N. (2011). The androgen receptor fuels prostate cancer by regulating central metabolism and biosynthesis. EMBO J..

[61] Juras P.K., Racioppi L., Mukherjee D., Artham S., Gao X., Akullian D’Agostino L., Chang C.Y., McDonnell D.P. (2023). Increased CaMKK2 Expression Is an Adaptive Response That Maintains the Fitness of Tumor-Infiltrating Natural Killer Cells. Cancer Immunol. Res..

