# Supplementary material for: CaMKK2 Expression Correlates with High-Risk CLL Biology, and Pharmacologic Inhibition Is Associated with Reduced Leukemic Cell Survival and Nurse-like Cell Support In Vitro
Source: Cells. 2026 Jul 20;15(14):1294. doi: 10.3390/cells15141294 (PMC13406266; doi:10.3390/cells15141294)
Supplement: Supplementary file 1 [file cells-15-01294-s001.zip › cells-4373433-supplementary.pdf]

Supplementary files

CaMKK2 Expression Correlates with High-Risk CLL Biology, and Pharmacologic Inhibition Is Associated with Reduced Leukemic Cell Survival and Nurse-Like Cell Support In Vitro

Supplementary Table S1. Demographic and clinical summary of CLL patient samples used for association studies (Figure 1).

	Overall	IGHV mutated	IGHV unmutated	CaMKK2 cohort
N	40	20	20	33*
Age at Dx, median [IQR]	60.0 [55.0-68.0]	60.0 [56.8-68.0]	59.0 [53.0-66.0]	61.0 [57.0-68.0]
Female	10 (25.0%)	7 (35.0%)	3 (15.0%)	9 (27.3%)
Male	30 (75.0%)	13 (65.0%)	17 (85.0%)	24 (72.7%)
Rai 0	26 (65.0%)	19 (95.0%)	7 (35.0%)	24 (72.7%)
Rai 1–2	11 (27.5%)	1 (5.0%)	10 (50.0%)	6 (18.2%)
Rai 3–4	3 (7.5%)	0 (0.0%)	3 (15.0%)	3 (9.1%)
IGHV unmutated	20 (50.0%)	0 (0.0%)	20 (100.0%)	14 (42.4%)
CD38 ≥30%	10 (25.0%)	1 (5.0%)	9 (45.0%)	8 (24.2%)
ZAP70 ≥20%	24 (60.0%)	10 (50.0%)	14 (70.0%)	18 (54.5%)
13q del (any)	20 (50.0%)	12 (60.0%)	8 (40.0%)	16 (48.5%)
11q del (any)	5 (12.5%)	1 (5.0%)	4 (20.0%)	5 (15.2%)
17p del (any)	2 (5.0%)	1 (5.0%)	1 (5.0%)	2 (6.1%)
trisomy 12 (any)	5 (12.5%)	1 (5.0%)	4 (20.0%)	4 (12.1%)
normal FISH	12 (30.0%)	6 (30.0%)	6 (30.0%)	10 (30.3%)
OS, months median [IQR]	159.8 [79.8-184.7]	186.3 [174.3–230.7]	79.3 [47.8-117.6]	164.1 [100.8-195.2]
TTT, months median [IQR]	87.4 [10.9-178.7]	178.8 [168.5–211.3]	10.7 [3.0-13.5]	159.6 [12.8-179.1]
Deaths	11 (27.5%)	5 (25.0%)	6 (30.0%)	8 (24.2%)

*Samples assessed for *CaMKK2* gene expression.

Supplementary Table S2. Demographic and clinical summary of CLL patient samples used for *in vitro* studies.

CLL ID#	TTT (Year)	Rai	IGHV Mut	CD38	ZAP70	FISH	FISH Good/Bad	Age at Dx	Sex	
69	15.6	0	-	+	+	13q	Good	62	male	Figure 2
400	2.53	0	+	-	-	13q	Good	48	male	
499	15.54	0	+	-	+	ND	ND	57	male	
558	5.25	ND	+	-	+	13q	Good	47	male	
700	2.45	0	+	+	-	13q	Good	69	male	
729	1.8	0	+	-	-	Normal	Good	66	male	
**763**	4.48	0	-	-	+	13q	Good	64	male	
866	0.5	1	ND	-	+	Normal	Good	66	male	
824	0.16	0	ND	-	+	11q13q	Bad	54	male	
338	N/A	0	-	-	+	13q	good	64	male	Figure S2
467	N/A	0	+	-	-	13q	good	71	female	
649	6.79		+	-	+	13q	good	53	male	
673	3.66	N/A	-	N/A	+	tri12	intermediate	53	male	
686	3.97	0	-	+	+	normal	good	61	female	
766	4	N/A	+	N/A	N/A	11q13q	bad	35	male	
768	N/A	0	N/A	-	-	13q	good	41	male	
783	N/A	0	N/A	N/A	N/A	13q	good	56	male	
842	N/A	0	+	-	-	13q	good	67	male	
846	11.94	0	+	N/A	N/A	tri12	intermediate	62	female	
862	N/A	1	-	+	N/A	normal	good	73	male	
46	2.19	0	-	-	+	13q	good	58	female	Figure 3A
183	13.66	0	+	-	-	13q	good	59	female	
490	8.63	0	+	-	-	normal	good	65	female	
631	5.3	ND	+	+	+	ND	ND	63	male	
649	6.79		+	-	+	13q	good	53	male	
716	4.62	0	-	-	+	13q	good	59	female	
**763**	4.48	0	-	-	+	13q	Good	64	male	
842	N/A	0	+	-	-	13q	good	67	male	
338	N/A	0	-	-	+	13q	good	64	male	Figure 3B
569	N/A	ND	+	-	-	13q	good	59	male	
649	6.79		+	-	+	13q	good	53	male	
723	N/A	0	+	-	-	13q	good	73	male	
766	4	N/A	+	N/A	N/A	11q13q	bad	35	male	
781	N/A	N/A	+	N/A	N/A	13q	good	71	male	
783	N/A	0	N/A	N/A	N/A	13q	good	56	male	
862	N/A	1	-	+	N/A	normal	good	73	male	
621	5.39	0	ND	-	-	13q	good	59	male	Figure 3C
647	N/A	0	+	-	-	13q	good	48	female	
649	6.79		+	-	+	13q	good	53	male	
733	0.08	N/A	N/A	-	N/A	17p13q	bad	68	female	
791	N/A	0	N/A	N/A	N/A	tri12	intermediate	56	male	
467	N/A	0	+	-	-	13q	good	71	female	Figure 3D
569	N/A	ND	+	-	-	13q	good	59	male	
649	6.79		+	-	+	13q	good	53	male	
673	3.66	N/A	-	N/A	+	tri12	intermediate	53	male	
686	3.97	0	-	+	+	normal	good	61	female	
714	11.18	0	+	N/A	N/A	13q	good	52	male	
768	N/A	0	N/A	-	-	13q	good	41	male	
783	N/A	0	N/A	N/A	N/A	13q	good	56	male	
842	N/A	0	+	-	-	13q	good	67	male	
862	N/A	1	-	+	N/A	normal	good	73	male	
166	N/A	0	+	-	+	normal	good	44	female	Figure 4*
400	2.53	0	+	-	-	13q	Good	48	male	
791	N/A	0	N/A	N/A	N/A	tri12	intermediate	56	male	

*CLL686 was also be used to generate data in Figure 4C.

Supplementary Table S3. List of primers used for qRT-PCR gene expression analysis.

Gene	Forward [5'-3']	Reverse [5'-3']
CaMKK2	AGC TGA GGA CTT GAA GGA CCT GAT	AGG TTG TCT TCG CTG CCT TGC TT
IL6	AAC CTG AAC CTT CCA AAG ATG G	TCT GGC TTG TTC CTC ACT ACT
CXCL10	GTG GCA TTC AAG GAG TAC CTC	GCC TTC GAT TCT GGA TTC AGA CA
APRIL	CTC TGC TGA CCC AAC AAA CAG	TTT TCC GGG ATC TCT CCC CAT
BAFF	GGG AGC AGT CAC GCC TTA	CGT GGG AGG ATG GAA ACA CAC
ACTB	CCT TGC ACA TGC CGG AG	GCA CAG AGC CTC GCC TT

Supplementary Table S4. Multivariable linear regression of CaMKK2 expression with IGHV mutation status and Rai stage (Duke CLL cohort).

Predictor	Beta_log10	CI95-low	CI95-high	Fold change-10^beta	p
IGHV unmutated (vs mutated)	0.4960	0.3102	0.6818	3.1330	1.6766e-07
Rai stage, per 1 unit	-0.0565	-0.1647	0.0518	0.8781	0.3068

Supplementary Table S5

	Patient	Patient	Patient
**Drug EC50 (uM)**	**621-007**	**647-011**	**733-012***
SGC-CaMKK2-1	5.29	5.49	27.71
CC-8977	28.56	14.66	212.45
STO-609	13.46	7.16	36.42

*Multi-drug-resistant patient.

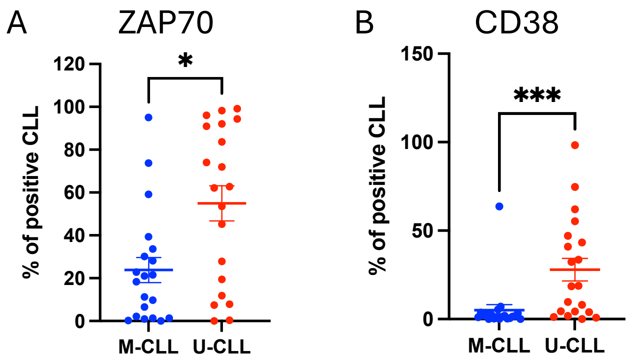

Figure S1. Canonical CLL prognostic markers CD38 and ZAP-70 are increased in IGHV-unmutated cases. Flow cytometry was performed on peripheral blood $CD19^{+}$ CLL cells to quantify ZAP-70 (A) and CD38 (B) expression. Samples were stratified by IGHV mutation status (M-CLL vs U-CLL). Each symbol represents one patient; horizontal bars indicate mean ± SEM. Group differences were assessed using the Mann-Whitney test; significance is indicated by asterisks.

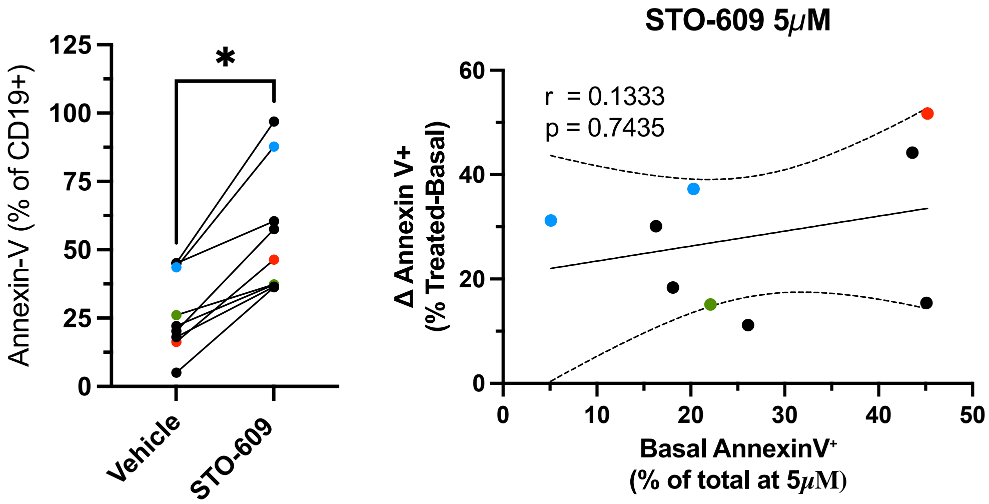

Figure S2. CaMKK2 inhibition increases apoptosis in primary CLL cells independently of basal apoptosis levels. Primary CLL cells isolated from fresh PBMC samples were treated with STO-609 or vehicle control. (A) Flow-cytometry analysis of Annexin V positivity showed increased apoptosis after treatment with 5 μM STO-609 compared with vehicle control. Each paired point represents an independent patient sample. (B) The STO-609-induced increase in apoptosis, calculated as ΔAnnexin V positivity relative to matched vehicle-treated cells, was plotted against basal apoptosis levels measured in vehicle-treated cells. No significant association was observed between basal apoptosis and the magnitude of STO-609-induced apoptosis, suggesting that the pro-apoptotic effect of CaMKK2 inhibition is not simply explained by higher pre-existing cell death. Associations were assessed by Spearman rank correlation; regression line is shown for visualization. *$p < 0.05$.

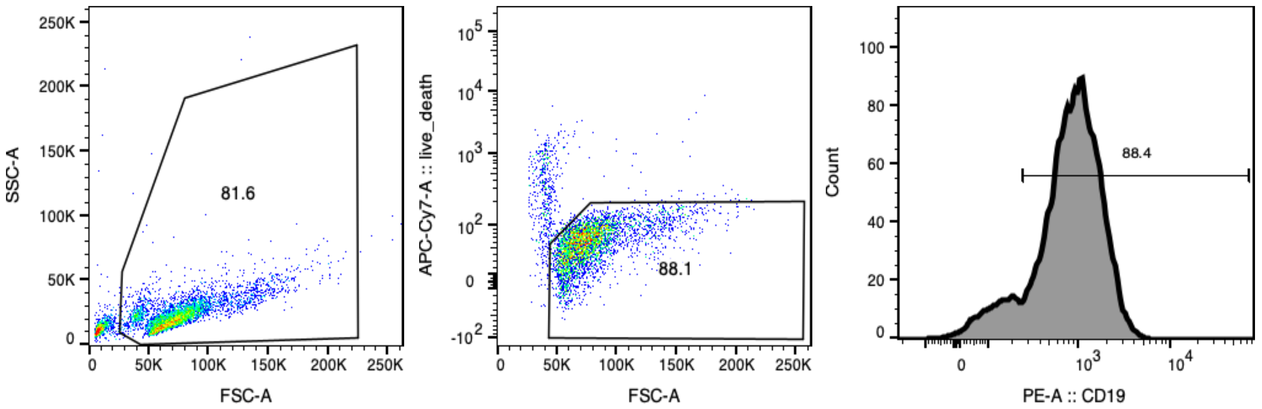

Figure S3. Gating strategy and baseline phenotype of a representative PBMC-CLL sample at day 0 of long-term culture. Representative flow-cytometry plots of a PBMC-CLL sample at day 0 of long-term culture. The left panel shows the initial cell gate based on forward and side scatter properties. The middle panel shows identification of viable cells using live/dead staining, based on the left panel gate. The right panel shows CD19 expression within the live-cell gate. Numbers indicate the percentage of events within the indicated gates. The CD19 gate was set using the fluorescence-minus-one (FMO) control.

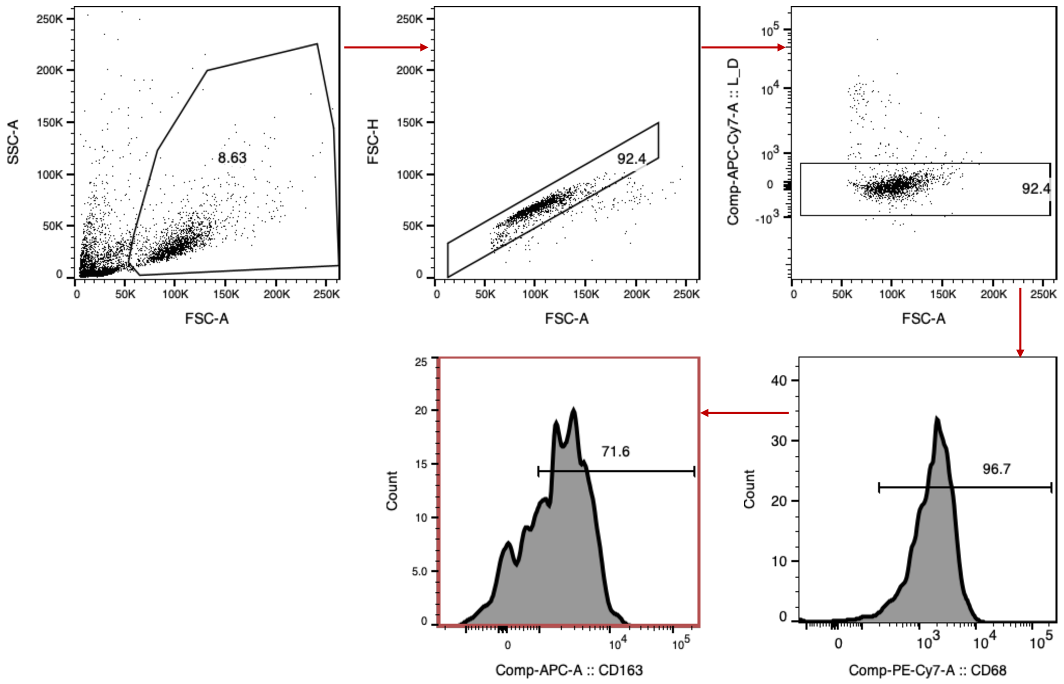

Figure S4. Flow cytometry gating strategy for identification of CD163⁺ macrophages. Representative flow cytometry plots illustrating the sequential gating strategy used to identify CD163⁺ macrophages derived from peripheral blood mononuclear cell (PBMC) of CLL and healthy donors. Detached adherent cells were first gated based on forward and side scatter (FSC-A/SSC-A) parameters to exclude debris and select the viable cell population. Doublets were excluded using FSC-area versus FSC-height gating (FSC-A/FSC-H). The macrophage population was then defined based on CD68 expression. Within this gated CD68⁺ compartment, CD163 expression was assessed to quantify the CD163⁺ macrophage subset. Histograms show representative CD163 staining with corresponding gating thresholds. Percentage values indicate the proportion of CD163⁺ cells within the CD68⁺ macrophage population. Gates were defined using fluorescence minus one (FMO) controls where applicable.

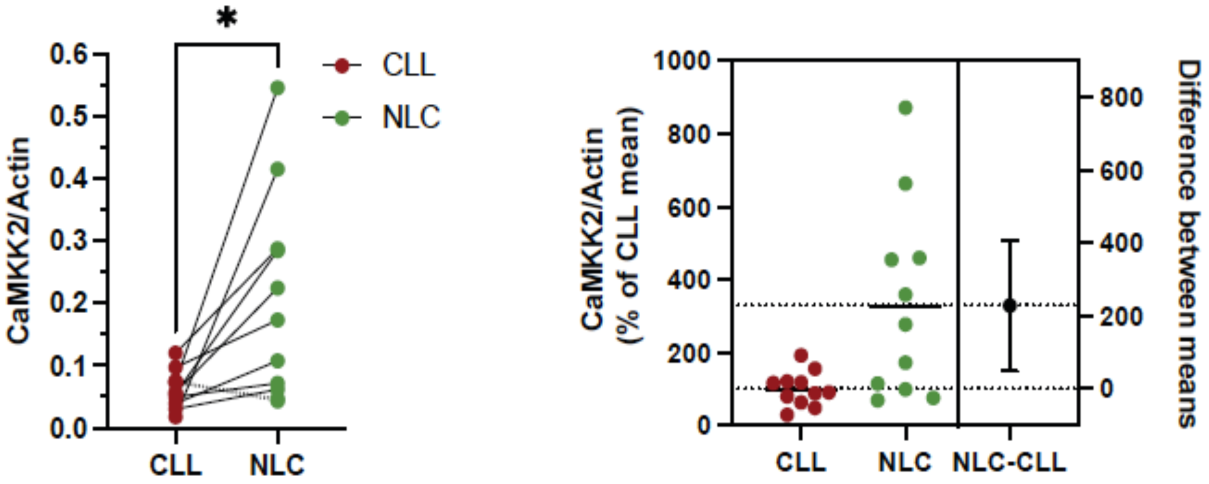

Figure S5. ***CaMKK2*** **expression in CLL and corresponding autologous NLC.** CaMKK2 expression in CLL cells and paired autologous NLC. PBMCs from CLL patients were cultured at high density for 14 days to generate a non-adherent $CD19^+$ CLL fraction and an adherent NLC-enriched fraction. Left: CaMKK2 mRNA expression (CaMKK2/ACTB) measured by qRT-PCR in purified $CD19^+$ CLL cells (CLL) and matched autologous adherent NLC; each line connects paired measurements from the same patient. Statistical significance was assessed using a two-tailed paired Wilcoxon signed-rank test; $p < 0.05$. Right: Estimation plot showing CaMKK2 expression in CLL and NLC, and the paired difference (NLC-CLL), expressed as a percentage of the mean CaMKK2/ACTB value in the CLL group; points represent individual patients and error bars indicate the mean difference with 95% confidence interval.
